# Functional Characterization of Sugar Transporter CRT1 Reveals Differential Roles of Its C-Terminal Region in Sugar Transport and Cellulase Induction in Trichoderma reesei

**DOI:** 10.1128/spectrum.00872-22

**Published:** 2022-07-19

**Authors:** Zhixing Wang, Renfei Yang, Wenhao Lv, Weixin Zhang, Xiangfeng Meng, Weifeng Liu

**Affiliations:** a State Key Laboratory of Microbial Technology, Microbiology Technology Institute, Shandong University, Qingdao, People’s Republic of China; University of Natural Resources and Life Sciences Vienna

**Keywords:** *Trichoderma reesei*, cellulase induction, Crt1, transceptor, transporter

## Abstract

The expression of cellulase genes in lignocellulose-degrading fungus Trichoderma reesei is induced by insoluble cellulose and its soluble derivatives. Membrane-localized transporter/transceptor proteins have been thought to be involved in nutrient uptake and/or sensing to initiate the subsequent signal transduction during cellulase gene induction. Crt1 is a sugar transporter proven to be essential for cellulase gene induction although the detailed mechanism of Crt1-triggered cellulase induction remains elusive. In this study, we focused on the C-terminus region of Crt1 which is predicted to exist as an unstructured cytoplasmic tail in *T. reesei*. Serial C-terminal truncation of Crt1 revealed that deleting the last half of the C-terminal region of Crt1 hardly affected its transporting activity or ability to mediate the induction of cellulase gene expression. In contrast, removal of the entire C-terminus region eliminated both activities. Of note, Crt1-C5, retaining only the first five amino acids of C-terminus, was found to be capable of transporting lactose but failed to restore cellulase gene induction in the Δ*crt1* strain. Analysis of the cellular localization of Crt1 showed that Crt1 existed both at the plasma membrane and at the periphery of the nucleus although the functional relevance is not clear at present. Finally, we showed that the cellulase production defect of Δ*crt1* was corrected by overexpressing Xyr1, indicating that Xyr1 is a potential regulatory target of the signaling cascade initiated from Crt1.

**IMPORTANCE** The lignocellulose-degrading fungus *T. reesei* has been widely used in industrial cellulases production. Understanding the precise cellulase gene regulatory network is critical for its genetic engineering to enhance the mass production of cellulases. As the key membrane protein involved in cellulase expression in *T. reesei*, the detailed mechanism of Crt1 in mediating cellulase induction remains to be investigated. In this study, the C-terminal region of Crt1 was found to be vital for its transport and signaling receptor functions. These two functions are, however, separable because a C-terminal truncation mutant is capable of sugar transporting but loses the ability to mediate cellulase gene expression. Furthermore, the key transcriptional activator Xyr1 represents a downstream target of the Crt1-initiated signaling cascade. Together, our research provides new insights into the function of Crt1 and further contributes to the unveiling of the intricate signal transduction process leading to efficient cellulase gene expression in *T. reesei*.

## INTRODUCTION

Lignocellulose, as the most abundant and cost-effective sustainable resource, exhibits tremendous potential in the production of biofuel and bio-based chemicals (1). However, due to the recalcitrant nature and complex structure of lignocellulose, there are inherent challenges in its bioconversion (2). Known for its high capacity to secrete cellulases, the filamentous fungus Trichoderma reesei has been used as the principal microorganism for industrial cellulase production (3, 4). While stringently controlled by catabolite repression, the expression of cellulase genes in *T. reesei* can be rapidly induced by either insoluble crystalline cellulose or soluble substrates, such as cellobiose, sophorose, and lactose (5). Regarding the induction of cellulases by insoluble cellulose, it is generally acknowledged that the basal level of cellulases produced by the fungus act on the insoluble substrate to release and even transform soluble inducers to facilitate cellulase gene expression (6, 7). However, the precise mechanism of how soluble inducers are perceived to initiate the signal transduction cascade leading to efficient cellulase gene expression in *T. reesei* is still enigmatic (2).

Membrane transporter or receptor proteins are important components for mediating cell-environment communication, including sensing and transmitting nutritional signals (8). While some signaling substrates can be directly taken up into the cell by transporters and further participate in the subsequent intracellular signaling process, most ligands usually bind signaling receptors at cell surface without further intracellular delivery of the ligand (9). The conformational changes induced by ligand binding lead to intracellular signaling cascades usually initiated by recruiting specific protein components on the intracellular tail region of the receptor. More recently, another family of membrane proteins has been found to be capable of simultaneously transporting substrates and carrying out the corresponding signaling functions (8, 9). Notably, over 100 transporter-encoding genes are annotated in the *T. reesei* genome and some of them have been verified to be capable of transporting diverse substrates, including monosaccharides and disaccharides (10–14). For instance, Stp1 was identified to transport both cellobiose and glucose, and its absence enhanced cellulase production (12). Importantly, two cellodextrin transporters in Neurospora crassa, CDT-1 and CDT-2, have been found to be responsible for the efficient induced expression of cellulase genes. However, it was later demonstrated that CDT’s function in mediating cellulase gene induced expression is not absolutely dependent on its transporting activities (15, 16). Similar to N. crassa, a major facilitator superfamily (MFS) sugar transporter Crt1 has been identified to be essential for the induced cellulase production in *T. reesei* when cultured on lactose or Avicel (12, 17). Recent studies verified that like CDT-1 and CDT-2, Crt1 is capable of transporting lactose, cellobiose, and sophorose (14, 18). However, it remains unresolved whether the essential role of Crt1 in cellulase gene expression is intricately dependent on its transporting activities.

In this study, a dual cellular localization of Crt1 was detected. Furthermore, the functional significance of *T. reesei* Crt1 in transport and signaling was emphasized by constructing different C-terminally truncated mutants of Crt1. These two functions were, however, found to be separable in Crt1. Our results thus revealed some novel characteristics of Crt1 in mediating the efficient induced expression of *T. reesei* cellulase genes.

## RESULTS

### Crt1 is localized at the cell membrane as well as at the periphery of the nucleus.

**FIG 1:**
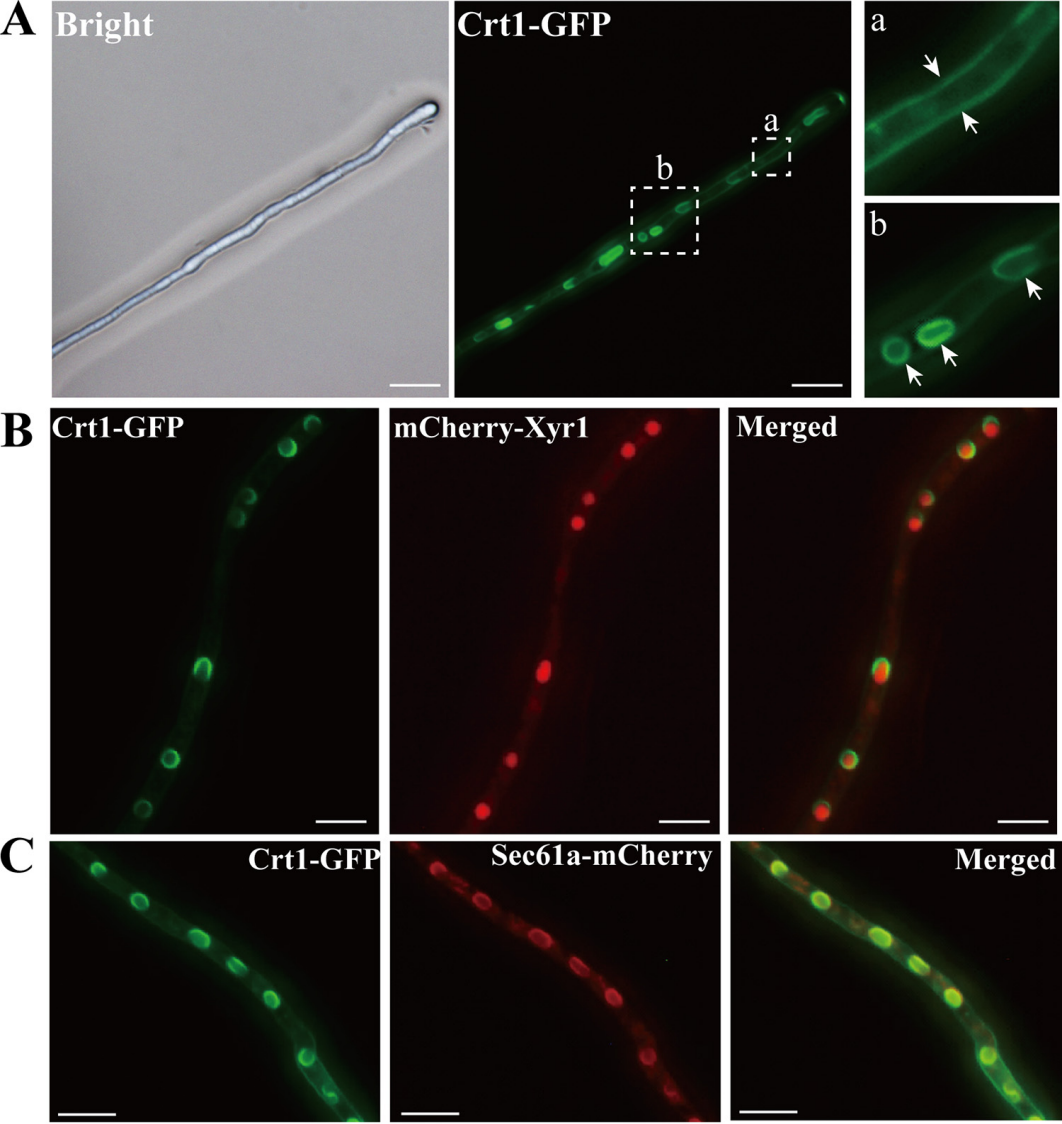
Subcellular localization of Crt1-GFP under cellulase induction condition. (A) Fluorescence microscopy analysis of Crt1-GFP after inducing with 1% (wt/vol) Avicel in MA medium for 12 h. Inset a: arrows indicated the plasma membrane; Inset b: arrows indicated the nuclear envelope. Scale bar: 10 μm. (B) Colocalization of Crt1-GFP and mCherry-Xyr1 after inducing with 1% (wt/vol) Avicel for 12 h. Scale bar: 10 μm. (C) Colocalization analysis of Crt1-GFP and the NE-localized Sec61α-mCherry. The strain was cultured in MA medium with 1% (wt/vol) Avicel for 12 h and mycelia were collected for fluorescence analysis. Scale bar: 10 μm. The fluorescence was examined with a Nikon Eclipse 80i fluorescence microscope. The images shown are taken from one of at least two independent experiments.

The location of membrane proteins may have important indications on their functions. To examine the precise subcellular localization of Crt1, we constructed the Crt1-green fluorescent protein (GFP) strain by fusing the *gfp* to the C-terminus of the endogenous *crt1* (Fig. S1). Hardly any difference in the extracellular total protein concentration and *p*-nitrophenyl-β-d-cellobioside (*p*NPC) hydrolytic activities was detected between the Crt1-GFP and wild-type strains (Fig. S2), indicating that Crt1-GFP is functionally equivalent to wild type Crt1. The fluorescence signal of Crt1-GFP was readily detected on the plasma membrane under inducing conditions (Avicel, lactose, sophorose, and cellobiose) (Fig. 1A; Fig. S3A). Of note, fluorescence signal also appeared inside the hypha and displayed a semicircular or circular distribution (Fig. 1A; Fig. S3A). As a comparison, Stp1, another identified sugar transporter involved in *T. reesei* cellulase expression (12), was shown to be exclusively located at the cell membrane under Avicel condition (Fig. S3B).

To check the potential cellular localization of Crt1, we analyzed the colocalization of Crt1 and nuclear-localized Xyr1, the key transcriptional activator for cellulase genes (19, 20). As shown in Fig. 1B, Crt1-GFP signal was found to locate at the periphery of mCherry-Xyr1 signal, implicating that Crt1-GFP was located on the nuclear envelope (NE). Crt1-GFP was also found to colocalize with Sec61α-mCherry, an ER protein-conducting channel component (Fig. 1C). Further cellular fractionation followed by Western blotting assay confirmed the presence of Crt1-GFP in plasma membrane and outer nuclear membrane fractions (Fig. S4). Taken together, Crt1 displayed a dual cellular localization although the functional relevance of this localization remains unknown.

### The role of C-terminal part of Crt1 in its transporting activity.

Structure prediction of Crt1 (TrireRUTC30_1:109243) by TMHMM suggests that Crt1 contains 12 transmembrane domains, with a relatively long intracellular central loop, an N-terminus with 46 amino acids, and a C-terminus with 44 amino acids (Fig. S5). Considering the importance of the C-terminus of many receptors in signal transduction and endocytosis (21–24), we *in situ* deleted the predicted C-terminus of Crt1. The resultant mutant strain completely lost the ability to biosynthesize cellulases, a phenotype identical to that of Δ*crt1* (Fig. S2), verifying the importance of the C-terminus for Crt1 function.

**FIG 2:**
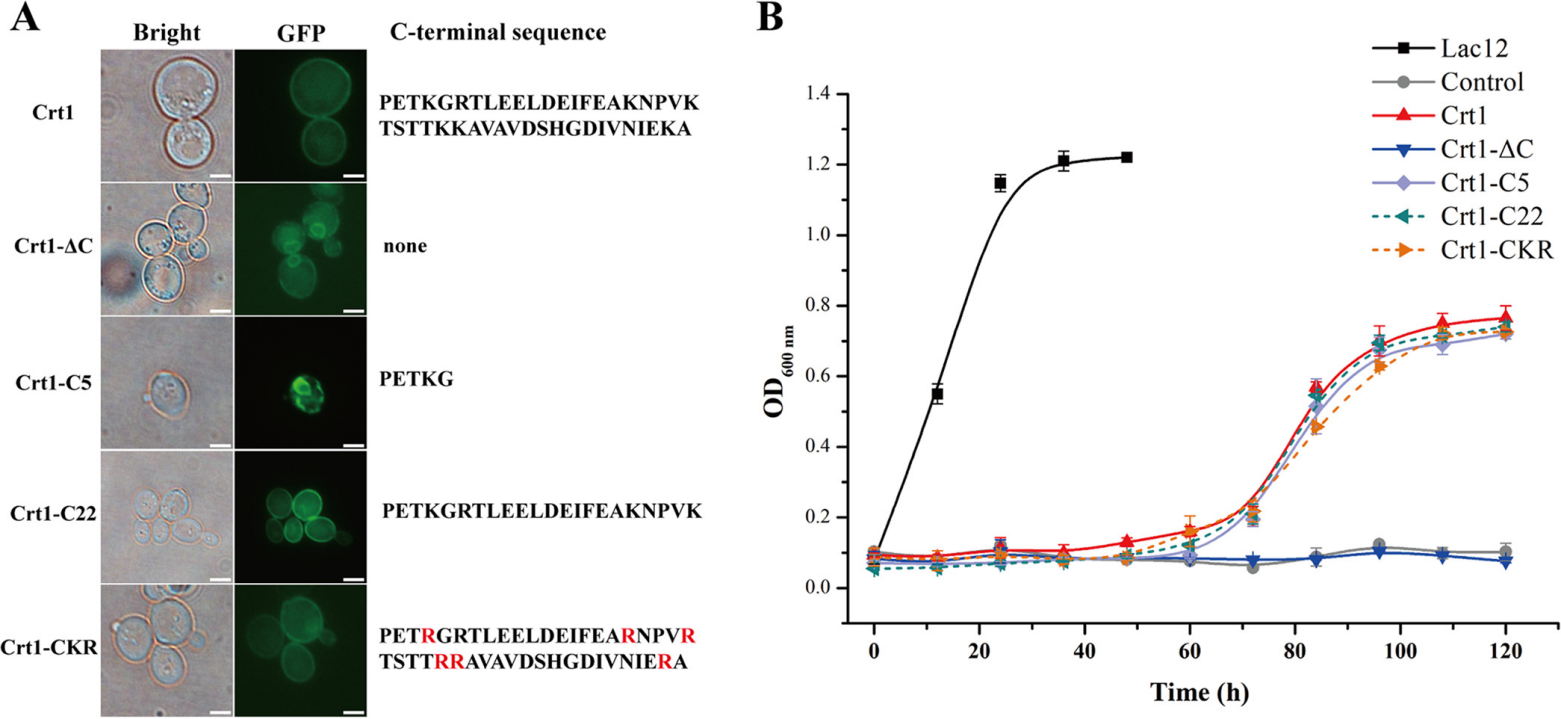
Lactose transport activity analysis of wild type Crt1 and its C-terminal mutants. (A) Intracellular fluorescence analysis of wild type Crt1 and its different mutants in yeast cells. GFP was fused at the C-terminus of Crt1 and its mutants. The C-terminus sequence of each mutant was shown on the right. Images shown represent one of at least two independent experiments. Scale bar: 2 μm. (B) The growth (OD600_nm_) of engineered S. cerevisiae W303 strains expressing wild-type Crt1 or its mutants in SC medium with 1% (wt/vol) lactose as the carbon source. S. cerevisiae W303 strains were equipped with the intracellular β-galactosidase LAC4 from K. lactis before expressing Crt1 or its mutants. The lactose permease Lac12 from K. lactis was used as the positive control (black square) and the empty vector as the negative control (gray dot). The results shown in this figure are the mean of three biological replicates.

It has been recently reported that Crt1 is capable of transporting relevant disaccharides based on the corrected annotation of the Crt1 sequence (18). In order to investigate whether the C-terminal region of Crt1 is also involved in its transporting activities, we performed a serial truncation from the extreme C-terminus of Crt1 (Fig. 2A). Considering the importance of lysine residues in the endocytosis and internalization of receptor proteins (21, 25–27), a Crt1-CKR mutant wherein all 6 lysine residues in the C-terminal tail substituted by arginine was also constructed.

To analyze the lactose transporting activities of the constructed Crt1 mutants, a yeast system incorporating a β-galactosidase (Kluyveromyces lactis LAC4) was used, similar to the system used for the analysis of cellobiose transporting activity of Stp1 (12). The lactose permease LAC12 of K. lactis was employed as a positive control (28–30). Each Crt1 mutant protein was fused with GFP at the C-terminus and fluorescence signals could be readily detected for all mutants in engineered S. cerevisiae strains (Fig. 2A). Growth assays of the engineered yeast strains showed that LAC12 enabled a fast growth of the yeast strain on lactose-containing minimal medium. Wild-type Crt1 also enabled the growth of the engineered yeast strain although a relatively lower final biomass was obtained after a long lag time (Fig. 2B). All Crt1 mutants except Crt1-ΔC allowed a comparable yeast growth to the wild-type Crt1. Particularly, while Crt1-ΔC with the removal of the entire C-terminus lost its ability to transport lactose, Crt1-C5 that retains only the first five amino acids of the C-terminus was still transport capable.

### The transporting and signaling functions of Crt1 are separable.

**FIG 3:**
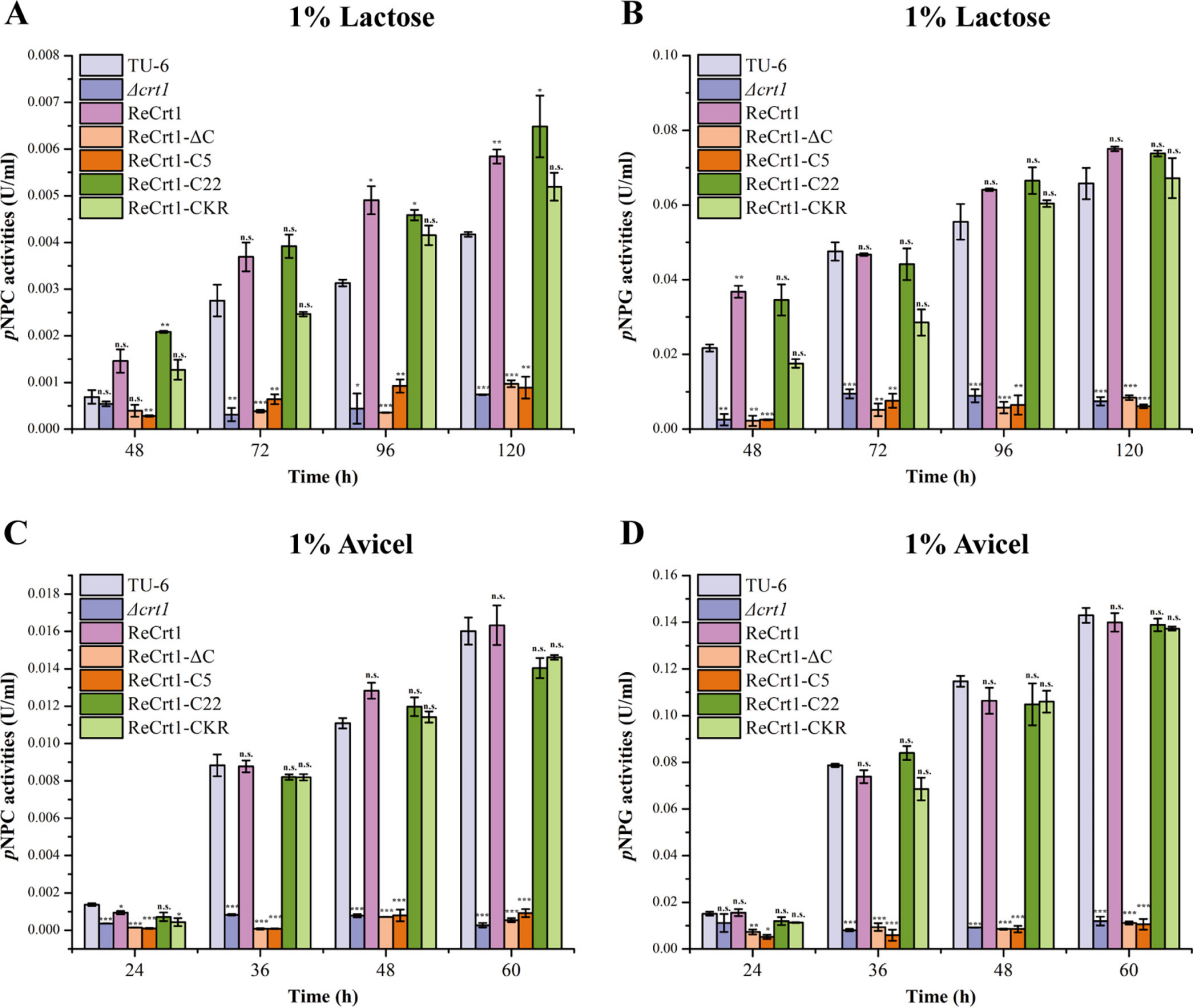
Functional complementation test of Crt1 C-terminal mutants in the Δ*crt1* strain. Extracellular *p*NPC hydrolytic activities (A and C) and *p*NPG hydrolytic activities (B and D) of supernatants from the parental strain TU-6, Crt1 deletion strain Δ*crt1* and different complementation strains cultured in MA medium containing 1% (wt/vol) lactose (A and B) or Avicel (C and D) as the sole carbon source for indicated time periods were determined. The wild type Crt1 and its mutants were expressed under the P*tcu1* promoter in their corresponding complementation strains. The values shown in this figure are the mean of three biological replicates. Error bars represent the standard deviation (SD) of these replicates. Statistical significances of extracellular activities relative to wild type TU-6 were determined using Student’s *t* test (n.s., *P* > 0.05; *, *P* < 0.05; **, *P* < 0.01; ***, *P* < 0.001).

To further investigate whether the necessity of Crt1 in mediating cellulase induction absolutely depends on its transporting activities, the competence of the constructed Crt1 mutants in inducing cellulase production was evaluated by complementing the Δ*crt1* strain with the wild-type and mutant Crt1 under the control of a copper responsive promoter P*tcu1* (31). Similar to wild-type Crt1, all Crt1 mutants displayed a dual-localization pattern (Fig. S6). As expected, wild-type Crt1 restored the cellulase production of Δ*crt1* on lactose and Avicel (Fig. 3). The extracellular *p*NPC and *p*-nitrophenyl-β-d-glucopyranoside (*p*NPG) hydrolytic activities of the complemented strain (ReCrt1) were even higher than those of the wild-type strain on lactose (Fig. 3A and B). Besides wild-type Crt1, complementation by Crt1-C22 and Crt1-CKR also rescued the impaired cellulase production of Δ*crt1* (Fig. 3). However, Crt1-ΔC failed to restore the cellulase secretion of Δ*crt1*, indicating the importance of the C-terminus in maintaining both transporting and signaling activities of Crt1. Of note, the transporting-active mutant Crt1-C5 also failed to induce cellulase production, implicating that signaling function of Crt1 is not absolutely correlated with its transporting activity.

**FIG 4:**
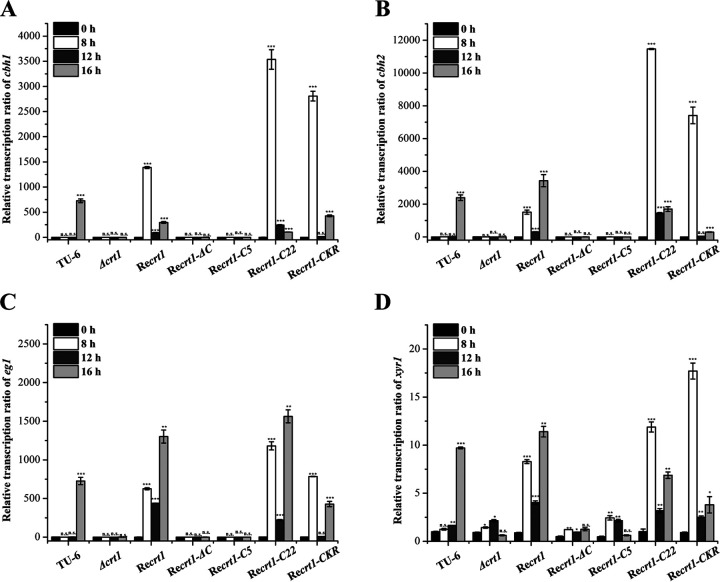
Transcriptional analysis of major cellulase genes and *xyr1* in TU-6, Δ*crt1*, and different complementary strains under lactose inducing condition. Relative transcription levels of main cellulase genes (*cbh1* [A], *cbh2* [B], and *eg1* [C]) and *xyr1* (D) were determined by quantitative RT-qPCR in a resting-cell system under 1% (wt/vol) lactose inducing condition. Values are the mean of three biological replicates and error bars represent the standard deviation (SD) of these replicates. The expression level of the actin gene was used as an endogenous control for all samples. Statistical significances of the target gene transcription relative to 0 h of each strain were determined using Student’s *t* test (n.s., *P* > 0.05; *, *P* < 0.05; **, *P* < 0.01; ***, *P* < 0.001).

The transcription of cellulase genes in different complemented strains was further analyzed in a resting-cell system. Remarkable upregulation in the transcription of the main cellulase genes (*chb1*, *cbh2*, and *eg1*) and the key transcriptional activator *xyr1* was detected at 8 h for ReCrt1, ReCrt1-C22, and ReCrt1-CKR strains, which is much earlier than the parental strain TU-6 (Fig. 4), indicating that constitutive expression of *crt1* facilitated the transcription of cellulase genes on lactose. In contrast, the mRNA levels of *cbh1*, *cbh2*, *eg1*, and *xyr1* remained undetectable in the ReCrt1-ΔC and ReCrt1-C5 strains. The induced transcription of cellulase genes was also detected in ReCrt1 strain under Avicel inducing conditions; however, the timing of cellulase gene transcription in ReCrt1 was not as advanced as that on lactose (Fig. S7), implicating a preference of Crt1 for soluble inducers when mediating the signaling process.

**FIG 5:**
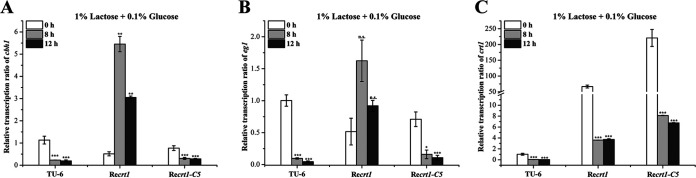
The carbon catabolite repression on the cellulase gene transcription was not fully relieved by overexpression of *crt1*. Transcriptional levels of *cbh1* (A), *eg1* (B), and *crt1* (C) in TU-6, Re*crt1*, and Re*crt1-*C5 strain were determined by quantitative RT-PCR at indicated time points in the resting-cell system using 1% lactose and 0.1% glucose as the carbon source. Values are the mean of three biological replicates and error bars represent the standard deviation (SD) of these replicates. The expression level of the actin gene was used as an endogenous control for all samples. Statistical significances of the target gene transcription relative to 0 h of each strain were determined using Student’s *t* test (n.s., *P* > 0.05; *, *P* < 0.05; **, *P* < 0.01; ***, *P* < 0.001).

To test whether the constitutive expressed Crt1 would contribute to the induced cellulase gene expression by antagonizing glucose-mediated catabolite repression, we analyzed the transcription of cellulase genes of ReCrt1, ReCrt1-C5, and TU-6 on 1% lactose and 0.1% glucose. Unlike TU-6 wherein cellulase gene expression was completely inhibited by the addition of 0.1% glucose, relatively low transcription was still detectable in ReCrt1 at 8 h and 12 h (Fig. 5A and B). Altogether these results confirmed that the C-terminus of Crt1 is implicated in the signaling function of Crt1, which is separable from its transporting activities.

### Overexpression of Xyr1 bypassed the cellulase production defect caused by *crt1* deletion.

**FIG 6:**
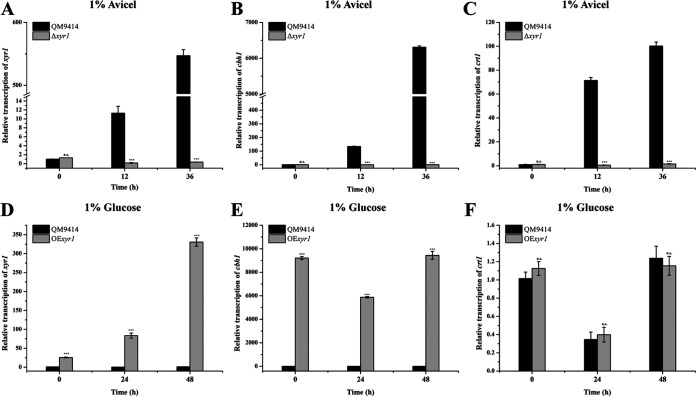
Xyr1 is essential but not sufficient for the transcription of *crt1*. The transcription levels of the endogenous *xyr1* (A or D), *cbh1* (B or E), and *crt1* (C or F) in QM9414, Δ*xyr1*, and OE*xyr1* strain were analyzed by quantitative RT-PCR at indicated time points. All strains were cultured in MA medium containing 1% (wt/vol) Avicel as the sole carbon source. Values are the mean of three biological replicates and error bars represent the standard deviation (SD) of these replicates. Statistical significances of the target gene transcription relative to wild type QM9414 were determined using Student's *t* test (n.s., *P* > 0.05; *, *P* < 0.05; **, *P* < 0.01; ***, *P* < 0.001).

Crt1 and Xyr1 represent the key upstream and downstream regulatory proteins involved in *T. reesei* cellulase induction, respectively. The functional connection between Crt1 and Xyr1 was further investigated. We found that the transcription of *crt1* is induced by Avicel in a similar pattern with cellulase genes (Fig. S8). Hence, we first verified whether the transcription of *crt1* is also activated by Xyr1. The transcription level of *crt1* was analyzed in QM9414 and the corresponding *xyr1* deletion strain (Δ*xyr1*). Similar to *cbh1*, the transcription of *crt1* was abolished in the absence of Xyr1 under Avicel inducing conditions (Fig. 6A to C), suggesting that Xyr1 is essential for the expression of *crt1*. We previously showed that overexpression of Xyr1 under the control of P*tcu1* enabled a constitutive cellulase expression even when the strain was cultivated with repressing carbon sources (31). The transcription of *crt1* was further analyzed in this strain on glucose. While the relative transcription level of *cbh1* in the OE*xyr1* strain was significantly upregulated compared with that in QM9414, no significant change was found in the mRNA level of *crt1* between OE*xyr1* and QM9414 (Fig. 6E and F). This result indicated that, unlike cellulase genes, overexpression of Xyr1 is not sufficient to promote the transcription of *crt1*. Taken together, our results revealed that Xyr1 is necessary but not sufficient for the expression of *crt1*.

**FIG 7:**
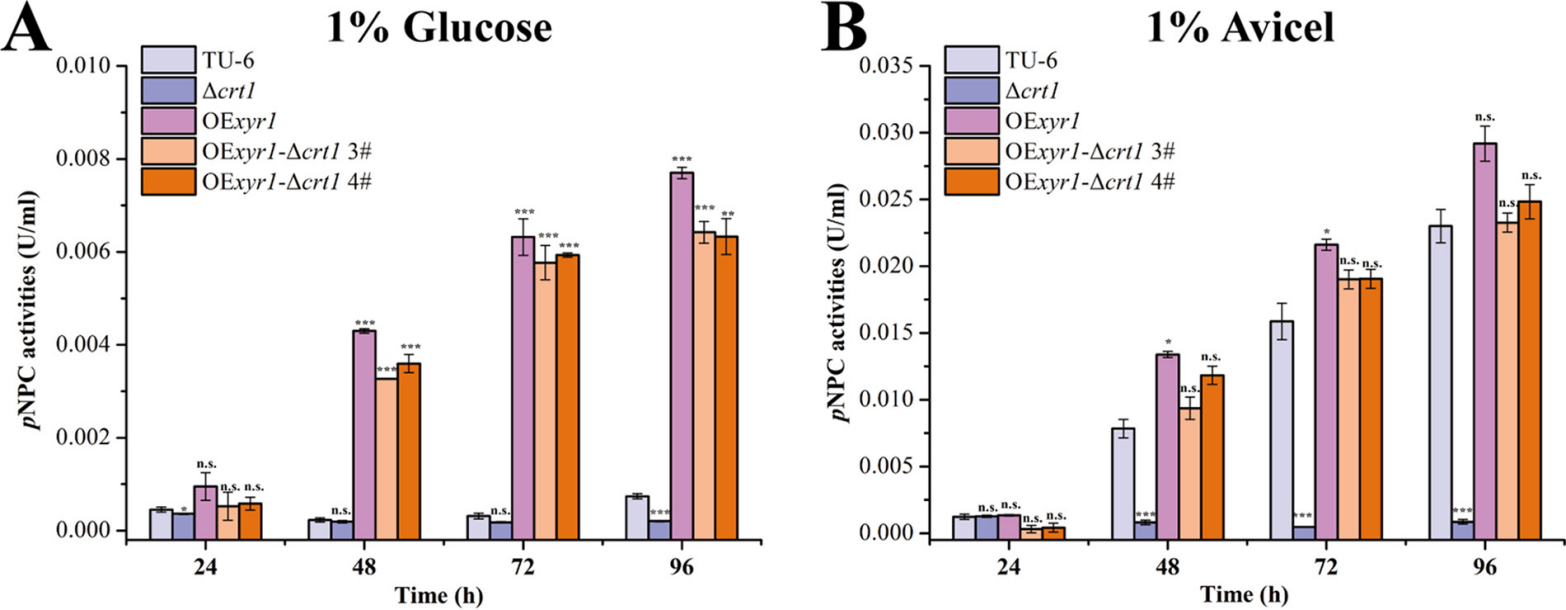
Overexpression of Xyr1 in Δ*crt1* bypassed its cellulase expression defect. Extracellular *p*NPC hydrolytic activities of supernatant from TU-6, Δ*crt1*, OE*xyr1*, OE*xyr1-*Δ*crt1* strains cultured in MA medium containing 1% (wt/vol) glucose (A) or 1% (wt/vol) Avicel (B) were determined. Values are the mean of three biological replicates and error bars represent the standard deviation (SD) of these replicates. Statistical significances of extracellular activities relative to wild type TU-6 were determined using Student's *t* test (n.s., *P* > 0.05; *, *P* < 0.05; **, *P* < 0.01; ***, *P* < 0.001).

As Crt1 seemed to be dispensable in the OE*xyr1* strain under glucose condition, we next explored whether the cellulase production defect of Δ*crt1* could be restored by overexpression of Xyr1. The absence of Crt1 in OE*xyr1* strain hardly affected the constitutive expression of cellulase genes when glucose was used as the sole carbon source (Fig. 7A). Specifically, the extracellular *p*NPC hydrolytic activities of the OE*xyr1*-Δ*crt1* strain were restored to the same level as the parental strain on Avicel (Fig. 7B). Taken together, our results implicate Xyr1 as a potential regulatory target of the Crt1-initiated signaling cascade.

## DISCUSSION

The cellulose-degrading fungus *T. reesei* is one of the most prominent cellulase producers in nature and is widely used as a workhorse for industrial cellulase production (4). Although the transcriptional regulation of cellulase gene expression has been intensively studied (20, 32–35), the precise mechanism of how *T. reesei* senses environmental nutrition cues to initiate intracellular signaling cascade remains largely unclear. Several fungal transporters have been shown to be involved in regulating cellulase expression. Until now, Crt1 is considered to be the most important transporter in *T. reesei* involved in cellulase induction. Its deletion completely abolished the expression of cellulase genes upon induction by cellulose and lactose (12, 17). Recently, Crt1 has been verified to be capable of transporting lactose, cellobiose, glucose, and sophorose (14, 18). However, it is still unclear to what extent the signaling function of Crt1 depends on its transporting activity to mediate cellulase gene induction.

Transporters and receptors located at the cell surface are considered to be functionally instrumental in ensuring the successful cellular communication with the environment not only in uptaking nutrition, but also in making rapid and appropriate response to changes in environment. In the present study, Crt1 was shown to locate at the plasma membrane as well as the nuclear periphery during the cellulase induction process. As reported, quite a few transporters and receptors have been found to be internalized from the plasma membrane and translocated to intracellular compartments such as endosomes, the Golgi apparatus, and nuclear membranes to mediate signal transduction (36). One prominent example is G-protein coupled receptors (GPCRs) that are endocytosed to be relocated to the nucleus. This relocalization is found to be capable of triggering identical or distinct signaling pathways as their counterparts on cell surface (37). On the other hand, the endoplasmic reticulum (ER) Sec61 translocon has been reported to localize at the nuclear membrane and assist the intracellular translocation of the epidermal growth factor receptor (EGFR) into the nucleus (38). Our results indicated an apparent colocalization between Sec61α-mCherry and Crt1-GFP. Unfortunately, we could not manage to get the knockout strain of Sec61α to dissect the functional significance of the observed colocalization. Alternatively, the observed internalization of Crt1 may just be a consequence of membrane proteins recycling to avoid their permanent membrane occupancy. Constitutively expressed Crt1-GFP was indeed also found to display a similar cellular localization under glucose or glycerol conditions, suggesting that the dual-localization of Crt1-GFP may not be correlated with the induction of cellulases. Whatever the case, the functional relevance of the observed dual-localization of Crt1 awaits further investigation.

The intracellular region, especially the C-terminus of many transporters and receptors, have been proved to be pivotal to their signaling functions although deletion of amino acids at the distal C-terminus did not affect transporting activities (21–24, 39, 40). For instance, removal of the entire C-terminus eliminated the transporting activity of human glucose transporter GLUT1 whereas removal of the last 21 amino acids had no effect (24). Our C-terminal truncation of Crt1 revealed that the Crt-C5 mutant with only the first five amino acids of the C-terminus domain retained the lactose transport activity, however, lost the ability to restore the cellulase gene expression of Δ*crt1* on lactose and Avicel. This result clearly demonstrated the involvement of Crt1 in *T. reesei* cellulase gene expression is correlated with its signaling function but not its transport activity. The transceptor roles in cellulase gene expression have also been demonstrated for CDT-1 and CDT-2 of N. crassa. Several mutants of CDT-1 and CDT-2 could hardly transport cellobiose but are still functional in triggering the transcription of cellulase genes (16). Our present study on Crt1 provided further evidence for the transporter protein acting as transceptors. Such mechanism may also exist in other fungi in regulating gene expression. Moreover, the Crt1-C22 mutant is functional in both transport and signal transduction, implying that the first half of the Crt1 C-terminus is sufficient for its transport and signal transduction activity. C-terminal phosphorylation of many receptors or transceptors was reported to be crucial for their functions. For example, the C-terminal tail of the yeast glucose sensor Rgt2 is phosphorylated by Ycks (yeast casein kinases) and this phosphorylation is required for corepressor binding and ultimately HXT gene expression (41). Studies on the ammonium transceptor Mep2 of S. cerevisiae showed that its activity was tightly regulated by the phosphorylation of S457 in its C-terminal region (42). For Crt1, we did detect some Crt1 related bands with a mobility shift that is different from the predicted molecular weight (Fig. S4), indicating that possible posttranslational modifications may occur on Crt1. However, the C-terminus of Crt1 (44 amino acids) is significantly shorter than those of Rgt2 (220 amino acids) and Mep2 (83 amino acids), and no canonical phosphorylation sites of casein kinases are predicted at the Crt1 C-terminus. On the other hand, ubiquitylation of lysine residues in the C-terminal region of some receptors has been shown to participate in their endocytosis and thus internalization (21, 25–27). Our results that substituting all six lysine residues in the C-terminal tail for arginine hardly affected Crt1 transporting or signaling activity suggested that ubiquitylation is not involved in regulating Crt1 activity. The precise mechanism by which Crt1 mediates the induced cellulase gene expression awaits further dissection.

Xyr1 is the key transcriptional activator of cellulase genes. While the absence of Xyr1 abolishes the transcription of all cellulase genes, including *crt1*, its overexpression has been reported to relieve the carbon catabolite repression (CCR) on cellulases expression (31). The transcription of *xyr1* was eliminated in Δ*crt1* on Avicel (12), implying that a positive feedback loop may exist between Crt1 and Xyr1 under Avicel inducing conditions. However, unlike cellulase genes, the transcription of *crt1* is maintained at a relatively low level when Xyr1 is overexpressed under glucose condition. This motivated us to investigate whether the phenotype of Δ*crt1* could be rescued by Xyr1 overexpression. As expected, overexpression of Xyr1 restored the cellulase production of Δ*crt1* to the same level of OE*xyr1* strain regardless of using Avicel or glucose as the sole carbon source. These results confirmed that Crt1 acts upstream of Xyr1 for activating cellulase gene expression. On the other hand, Crt1 overexpression enabled an earlier transcription peak of cellulase genes compared with the parental strain on lactose induction condition, a result consistent with those of Ivanova (17). However, the overexpression of Crt1 did not significantly relieve the CCR when a mixture of glucose (0.1%) and lactose (1%) was used as the carbon source. One possible explanation is that the signal cascade initiated from the constitutive expressed Crt1 could not completely override the CCR triggered by glucose to achieve the high-level expression of Xyr1, which finally lead to the cellulase gene expression. Moreover, Crt1 was also reported to transport glucose and the binding of lactose may be antagonized by glucose although the affinity for glucose is much lower than that of lactose (14). The observation that Xyr1 is essential but not sufficient for activating the expression of Crt1 implies that unique pathways may exist for the transcriptional regulation of *crt1*. Identification and interpretation of unknown transcriptional factors that act at the promoter of *crt1* will provide new insights into the global regulatory network of cellulase gene regulation.

## MATERIALS AND METHODS

### Strains, medium, and cultivation conditions.

Escherichia coli DH5α was used for plasmid construction and amplification. Strains were cultured in lysogeny broth in a rotary shaker at 37°C, 200 rpm. Saccharomyces cerevisiae W303-1A (*MATa*, *leu2-3*,*112*, *trp1-1*, *can1-100*, *ura3-1*, *ade2-1*, *his3-11*,*15*) was used for lactose transportation assay and was routinely grown in yeast extract-peptone-dextrose (YPD) medium (1% of yeast extract, 2% of peptone and 2% of glucose) at 30°C, 200 rpm. Uracil or leucine was left out in the synthetic complete (SC) medium (0.17% [wt/vol] yeast nitrogen base, 0.5% [wt/vol] ammonium sulfate, 2% [wt/vol] glucose) for transformant selection. SC medium with 2% (wt/vol) lactose instead of glucose as the carbon source was used for Crt1 transport activity test.

The *T. reesei* TU-6 (ATCC MYA-256), a uridine-auxotrophic derivative of *T. reesei* QM9414 (ATCC 26921), was used as a parental strain for recombinant strain construction in Crt1 function analysis (43). QM9414 (ATCC 26921) was used as parental strain for Δ*xyr1* and OE*xyr1* strain construction. All *T. reesei* strains were maintained on malt extract agar (Sigma-Aldrich, USA) supplemented with 10 mM uridine when necessary. For cellulase transcription and production analysis, *T. reesei* strains were precultured in 1-L Erlenmeyer flasks on a rotary shaker (200 rpm) at 30°C in 250 mL Mandels-Andreotti (MA) medium with glycerol (1%, vol/vol) as the carbon source for 36 h and transferred to the same fresh medium and further grown for another 12 h (44). Mycelia were harvested by filtration using the G1 funnel and washed twice with fresh MA medium with no carbon source. Equal amounts of mycelia were then transferred to a fresh MA medium containing 1% (wt/vol) lactose or Avicel (Sigma-Aldrich, USA), and cultivations were continued for the indicated time. Resting cell cultivations were performed as described previously (45). Briefly, strains were pregrown on glycerol medium and then washed extensively with the minimal medium lacking a carbon source. Washed mycelia were transferred to fresh medium with no carbon source and cultured at 30°C for 1 h to deplete intracellularly carbon and nitrogen nutrients. The mycelia were harvested and washed twice with 20 mM sodium citrate buffer (pH 5.0) and equal amounts of mycelia were then transferred to 250 mL of 20 mM sodium citrate supplemented with lactose (1%) or glucose (0.1%) as the carbon source. Mycelial samples were taken at indicated intervals for transcriptional analysis.

### Plasmids and strains construction.

To construct the Crt1-GFP strain, the complete coding region of *crt1* was amplified from genomic DNA, and fused with eGFP and the *trpC* terminator by fusion PCR. The resulting PCR fragment (*crt1*-eGFP-*trpC*) and 2.0 kb *crt1* downstream noncoding DNA fragment were ligated into pDONOR*pyr4* via BP-cloning (Invitrogen), yielding the *in situ* GFP-tagging vector pDONOR-Crt1-GFP, which was used to transform *T. reesei* after linearization with I-SceI. The pDONOR*pyr*4 plasmid was obtained as described previously (12).

For the colocalization analysis of Crt1-GFP and Sec61α, the coding regions of *sec61α* (Tr_121397) were amplified from genomic DNA and fused with mCherry coding sequence and *trpC* terminator at 3′ end. The generated DNA fragment and 2.0-kb downstream flanking sequence were digested with *Bam*HI/*Sma*I and *Hin*dIII/*Sal*I, respectively, and ligated into the corresponding sites of pUC*hph* sequentially to obtain pUC-Sec61a-mCherry, which were further linearized with *Ssp*I and transformed into Crt1-GFP strain. The pUC*hph* plasmid was obtained as described previously (45).

To construct the C-terminal truncated mutants, the transmembrane secondary structure of Crt1 was predicted by the TMHMM server (http://www.cbs.dtu.dk/services/TMHMM-2.0) and the C-terminal sequence of each mutant was indicated in Fig. 2A. The complete cDNA sequence of *crt1* was amplified from the total cDNA of TU-6 (induced with 1% [wt/vol] Avicel for 24 h) and further used as the template to amplify each C-terminal truncated mutant with designed primers. For Crt1-CKR mutant, the coding fragment was obtained by commercial gene synthesis (GENEWIZ, China). For expression of different Crt1 mutants under the control of P*tcu1* promoter in Δ*crt1* strain, the wild-type and mutant *crt1* were fused with eGFP at 3′ end. The generated DNA fragments were digested with *Asc*I and *Spe*I, and then ligated into the pMDP*tcu1*-T*trpC* plasmid (31). Each plasmid was cotransformed with plasmid pRLMex30 into Δ*crt1* strain (12, 46).

For the expression of Crt1 mutants in yeast, the cDNA of the wild-type and mutant *crt1* were amplified and ligated into the *Eco*RI/*Hin*dIII sites of pRS426ADH (12), in which the expression of Crt1 or its mutants was under the control of *ADH1* promoter. Expression of the β-galactosidase Lac4 and the lactose permease Lac12 were described previously (47).

Fungal transformation was performed as described by Penttila et al. (48). Transformants were selected on the minimal medium without uridine or minimal medium containing 120 μg/mL hygromycin. Anchored PCR was performed to verify the correct homologous integration events. All the primers used for plasmid construction and transformant verification were listed in Table S1.

### Microscopic analysis.

To visualize the fluorescence of Crt1-GFP and mutants-GFP fusion proteins in live cells, recombinant strains were typically prepared in the same way as for cellulase production analysis and samples were taken after culturing in MA medium for 12 h for microscopic analysis. Fungal hyphae were collected and spread onto glass slides and imaged with a Nikon Eclipse 80i fluorescence microscope (Nikon, USA) using a 60 × 1.4 NA oil immersion objective (Plan Apo VC). For colocalization analysis, filter sets for GFP (excitation 450 to 490 nm; emission 500 to 550 nm) or mCherry (excitation 538 to 562 nm; emission 570 to 640 nm) were used, respectively. All images were captured and processed using the NIS-ELEMENTSAR software.

### Protein extraction and Western blot analysis.

Strains were cultivated under Avicel-inducing conditions as described above. The membrane fraction was extracted by a previously reported method with some modifications (49). Briefly, mycelia were harvested by filtration, and the mycelial pads (1 to 2 g, wet weight) were ground with liquid nitrogen. Mycelia powders were suspended in 1 mL of extraction buffer (0.1 M Tris-HCl [pH 7.5], 0.15 M NaCl, 5 mM EDTA, plus a mixture of protease inhibitors [Complete, Roche], 1 mM phenylmethyl-sulfonyl fluoride [PMSF], and 25 mM freshly prepared N-ethylmaleimide) and kept on ice for 15 min. All subsequent steps were carried out at 4°C. Centrifugation at 1,500 *g* for 10 min was used to remove unbroken cells and debris and the supernatant was further centrifuged at 13,000 *g* for 60 min. The resulting pellet was resuspended in 400 μL of extraction buffer plus 5 M urea, incubated on ice for 1 h, and centrifuged at 13,000 *g* for 45 min. The pellets were then suspended in 320 μL extraction buffer plus 80 μL 50% trichloroacetic acid and incubated on ice for at least 30 min. Samples were centrifuged at 13,000 *g* for 45 min. The TCA protein precipitates were then neutralized in 25 μL 1 M Tris base plus 25 μL of 2× sample buffer (100 mM Tris-HCl, pH 6.8, 4 mM EDTA, 4% sodium dodecyl sulfate [SDS], 20% glycerol, 0.002% bromophenol blue, 2% β-mercaptoethanol) and heated at 37°C for 15 min. The obtained plasma membrane-bound proteins were analyzed by Western blotting.

Intact nuclei and the nuclear membrane fraction were isolated by modifying a method described by Adriana et al. (50). Mycelia were collected and ground in the same way used in membrane protein extraction. Mycelia powders were suspended in 5 mL cold TKMS buffer (0.1 M Tris-HCl [pH 7.5], 25 mM KCl, 5 mM MgCl_2_, 0.25 mM sucrose, plus a mixture of protease inhibitors [Complete, Roche]; 1 mM phenylmethyl-sulfonyl fluoride [PMSF]) and kept on ice for 15 min. All subsequent steps were carried out at 4°C. The homogenate was centrifuged at 500 *g* for 10 min to remove unbroken cells and debris. The supernatant was further centrifuged at 1,500 *g* for 15 min and the obtained pellet was washed twice with PBS buffer. The resulting nuclear preparations were resuspended in TDMS buffer (50 mM Tris-HCl [pH 7.4], 1 mM DTT, 10 mM MgCl2, 0.25 M sucrose) and considered as the intact nuclei fraction. Sodium citrate (1% wt/vol) was added to this nuclear suspension and incubated for 60 min on ice while stirring gently followed by centrifugation for 15 min at 500 *g*. The outer nuclear membrane in the supernatant was further precipitated by TCA as described above.

Western blot analysis was performed according to standard protocols (51). Detection of the Crt1-GFP was performed by immunoblot using a monoclonal antibody of GFP (Santa Cruz, USA).

### Yeast growth assay.

The constructed plasmids were transformed into S. cerevisiae W303 strain by the high-efficiency method of Gietz et al. (52). Selected transformants were grown in SC medium with 1% galactose under selective conditions and cells were harvested during logarithmic growth by centrifuging at 4,000 rpm for 5 min. The harvested cells were washed three times with water, adjusted to the same cell density of 0.1 (OD_600 nm_), and transferred to SC medium with 1% lactose as the sole carbon source. The growth curve was determined by measuring the optical density at 600 nm with Infinite M200 PRO (TECAN, Switzerland) and reported as the mean of three independent experiments.

### Enzyme activity and protein analysis.

The activities of cellobiohydrolases (*p*NPCase) and β-glucosidases (*p*NPGase) were determined by measuring the amount of released *p*-nitrophenol using *p*-nitrophenyl-β-d-cellobioside (*p*NPC, Sigma) and *p*-nitrophenyl-β-d-glucopyranoside (*p*NPG, Sigma) as substrates, respectively. The *p*NPC and *p*NPG activity assays were performed in 200-μL reaction mixtures containing 50 μL of culture supernatant and 50 μL of the respective substrate plus 100 μL of 50 mM sodium acetate buffer (pH 4.8) and then incubated at 50°C for 30 min. One unit (U) of *p*NPCase or *p*NPGase activity was defined as the release of 1 μmol pNP per minute under the test conditions (53). Total secreted proteins were determined using the Bradford protein assay with bovine serum albumin (BSA) as a standard. Three biological replicates were carried out for each experiment.

### Nucleic acid isolation and quantitative real-time PCR.

Fungal genomic DNA was extracted according to the instructions of E.Z.N.A. fungal DNA miniprep kit (Omega Biotech, USA). TRIzol reagent (Sangon Biotech, China) was used for total RNA isolation according to the manufacturer’s protocol and then a Turbo DNA-free kit (Ambion, USA) was used to digest and eliminate genomic DNA contamination. For reverse transcription (RT), a Hiscript III Reverse Transcriptase (Vazyme, China) was used according to the manufacturer’s instructions. Quantitative real-time PCRs (qRT-PCR) were performed by using *Taq* Pro Universal SYBR qPCR Master Mix (Vazyme, China) on a LightCycler 96 (Roche, Switzerland). Reactions were performed in triplicate with a total volume of 20 μL, including 250 nM (each) forward and reverse primers and template cDNA. Data were analyzed by using the relative quantitation/comparative threshold cycle (ΔΔCT) method and were normalized to an endogenous gene *actin* (35, 54–56). Three biological replicates were performed for each experiment.

### Statistical analysis.

Statistical analysis was performed using Student’s *t* test analysis. At least three biological replicates were performed for each analysis, and the results and errors are the mean and SD, respectively, of these replicates.

### Data availability.

All relevant data are included in the main body or the supplemental materials of this article.
