# Supplementary material for: Functional Characterization of Sugar Transporter CRT1 Reveals Differential Roles of Its C-Terminal Region in Sugar Transport and Cellulase Induction in Trichoderma reesei
Source: Microbiol Spectr. 2022 Jul 19;10(4):e00872-22. doi: 10.1128/spectrum.00872-22 (PMC9431493; doi:10.1128/spectrum.00872-22)
Supplement: Supplemental file 1 — Supplemental material. Download spectrum.00872-22-s0001.pdf, PDF file, 4.2 MB [file spectrum.00872-22-s0001.pdf]

Supplementary materials

Functional characterization of sugar transporter CRT1 reveals differential roles of its C-terminal region in sugar transport and cellulase induction in *Trichoderma reesei*

Zhixing Wang, Renfei Yang, Wenhao Lv, Weixin Zhang, Xiangfeng Meng*, Weifeng Liu

State Key Laboratory of Microbial Technology, Shandong University, Qingdao, People's Republic of China

Correspondence should be addressed to Xiangfeng Meng (email: x. meng@sdu.edu.cn).

Running title: Functional Roles of TrCRT1 in *T. reesei* Cellulase Induction

Table S1 Primers used in this study

Name	Sequence	Description
Crt1-up-F	TAGGGATAACAGGGTAATGAGATAGACATAAAAGCGGTAGCAC	Used for plasmid construction of the *in situ* fusion of GFP to the C-terminal of Crt1 to build Crt1-GFP strain.
Crt1-GFP-TrpC-R	TCGGGATCTTGCAGGCCGGGCGAGCCTTCTCGATATTGACAATGTCG	
GFP-TrpC-F	CATTGTCAATATCGAGAAGGCTCGCCCGGCCTGCAAGATCCCGAACG	
GFP-TrpC-R	GGGGACAAGTTTGTACAAAAAAGCAGGCTAATCGAGTGGAGATGTGGAGTGGGCGC	
Crt1-down -F	GGGGACCACTTTGTACAAGAAAGCTGGGTAGGCTTGGGGCTGTGAGCTGATGTTT	
Crt1-down-R	ATTACCCTGTTATCCCTAGAGGAAGATGCGGGTATCAGGAA	
Rab5A-m-up-F	TCCCCCGGGCCGCTCGAGAGGAAACCGTCTTCGTCTTCTGTCT	Used for plasmid construction of the *in situ* fusion of mCherry to the C-terminal of Rab5A.
Rab5A-m-up-R	CGCGGATCCCCATCGATGCAGGCACATCCATCCTTGGCGCCC	
Rab5A-m-down-F	ACGCGTCGACTTGGCGCGCCACGAGATTCGGGGTCTGTTGTGGCA	
Rab5A-m-down-R	CCCAAGCTTGGACTAGTTCATAGGAAACCCGCCAAAGGAA	
mcherry-TrpC-1-F	CCATCGATATGGTGAGCAAGGGCGAGGAGGATA	
mcherry-TrpC-1-R	TTTGATGATTTCAGTAACGTTAAGTCTACTTGTACAGCTCGTCCATGCCG	
mcherry-TrpC-2-F	CGGCATGGACGAGCTGTACAAGTAGACTTAACGTTACTGAAATCATCAAA	
mcherry-TrpC-2-R	CGCGGATCCTCGAGTGGAGATGTGGAGTGGGCGC	
Δend3-up-F	TCCCCCGGGCCGCTCGAGATGTCCCGTCTAGAATCGGAAGCAG	Used for plasmid construction of the *end3* deletion strain.
Δend3-up-R	TCCCCCGGGCAGCCCAGGGGAACCAAGGTATCGT	
Δend3-down-F	ACGCGTCGACATCTTGGGTGACTTTTTCACGCTTG	
Δend3-down-R	ATGGGCGGGAGCCAAGGAGAACAGC	
Sec61a-m-up-F	AAACGACGGCCAGTGAATTCGAGCTCAGTGGCTTAAACCTGGTTGGCGTGT	Used for plasmid construction of the *in situ* fusion of mCherry to the C-terminal of Sec61a.
Sec61a-m-up-R	ATTCTTATAATCTCTAGAGGATCCGGAATCTTAAGACCCATAATCATGCCCTTCATTCCG	
Sec61a-m-down-F	AGGAATAGACTAGTCCAATCGTCGACAGGGAGGCGATGGTTCTTTAA	
Sec61a-m-down-R	GCTATGACCATGATTACGCCAAGCTTGTTTAAACAGGCGTGAGATGGTCGTTGAT	
mcherry-TrpC-F	GGGCATGATTATGGGTCTTAAGATGGTGAGCAAGGCGAGGAGGATA	

mcherry-TrpC-R	GCATTCTTATAATCTCTAGAGGATCCTCGAGTGGAATGTGGAGTGGGCGC	
Ptcu1-Crt1-F	TTGGCGCGCCATGGGCGAGAAAGAAGACATTCA	Used for construction of the P*tcu1* driven expression of Crt1 mutants in *T.reesei Δcrt1*
Ptcu1-Crt1-R	AGCTTTGTTTAAACTAGCCTTCTCGATATTGACAATGTC	
Ptcu1-Crt1-ΔC-R	AGCTTTGTTTAAACTAATGAAAAAGTAGACGACAACCGTC	
Ptcu1-Crt1-C5-R	AGCTTTGTTTAAACTTCCCTTGGTCTCGGGAATGAAAAAG	
Ptcu1-Crt1-C22-R	AGCTTTGTTTAAACTCTTGACCGGGTTCTTGGCCTCG	
Ptcu1-Crt1-CKR-R	AGCTTTGTTTAAACTAGCCCTCTCGATATTGACAATGTCG	
pRS-Crt1-F	CCCAAGCTT ATGGGCGAGAAAGAAGACATTC	Used for assembling Crt1 mutants with GFP fused at their C-terminal to the yeast plasmid pRS426ADH1
pRS-GFP-R	GGAATTCTTAAGCCTTCTCGATATTGACAATG	
qcbh1-F	CTTGGCAACGAGTTCTCTT	Primers used for quantitative RT-PCR
qcbh1-R	TGTTGGTGGGATACTTGCT	
qcbh2-F	CGAATGGCGAATACTCTAT	
qcbh2-r	AGTCAGGCTCAATAACCA	
qeg1-F	CGGCTACAAAAGCTACTACG	
qeg1-R	CTGGTACTTGCGGGTGAT	
qxyr1-F	CCATCAACCTTCTAGACGAC	
qxyr1-R	AACCCTGCAGGAGATAGAC	
qcrt1-F	GATTCGGCGTCTCCATTG	
qcrt1-R	CGAGAACCAGAGAGTGTTG	
qactin-F	TGAGAGCGGTGGTATCCACG	
qactin-R	GGTACCACCAGACATGACAATGTTG	

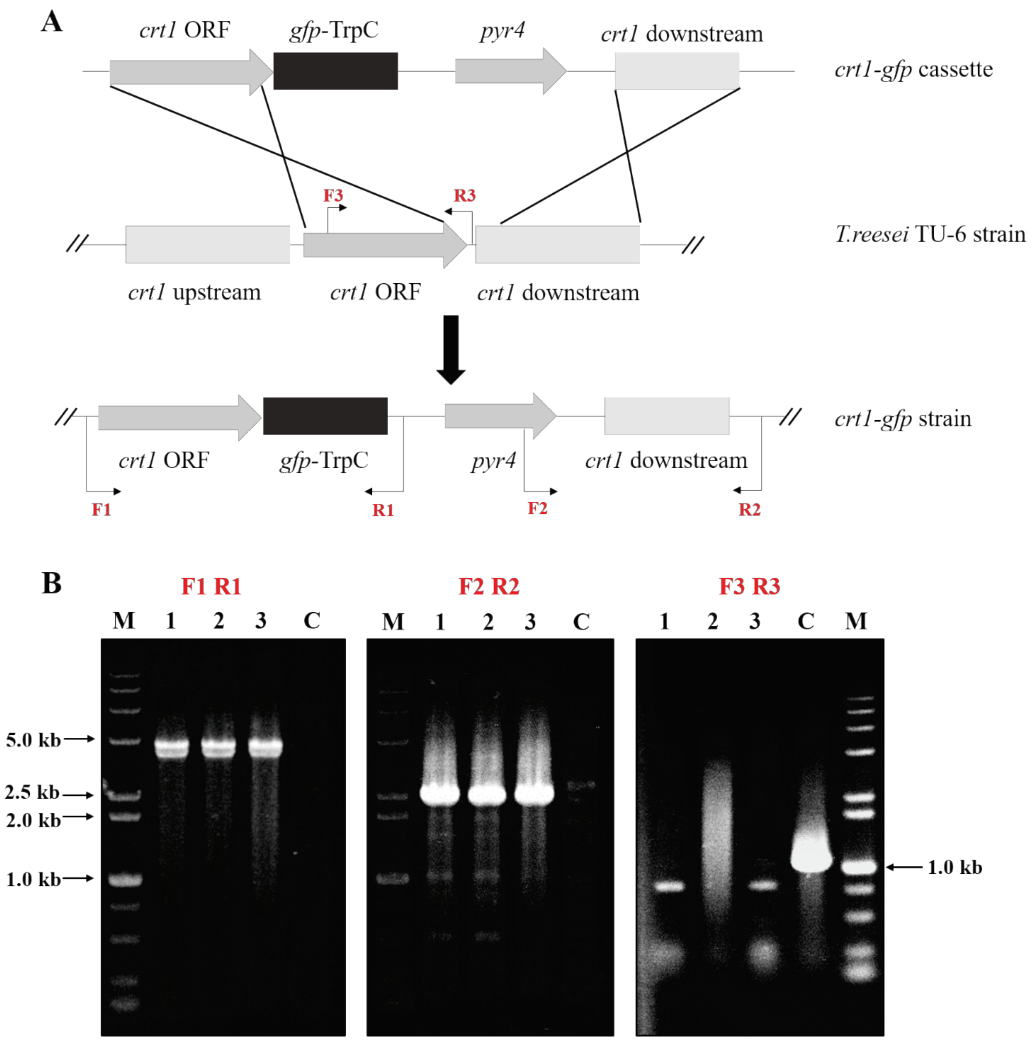

Figure S1. Schematic representation and verification of the construction of Crt1-GFP strain. (A) Schematic representation of the fusion of *crt1* and GFP *in situ* and illustration for verification of Crt1-GFP strain. (B) Diagnostic PCR was performed to verify the correct integration of the *crt1-gfp cassette*. The location of used primers was indicated in the figure. The genomic DNA (Lanes 1–3) from three independent transformants and the TU-6 genomic DNA (Lane C) was used as the template for PCR amplification.

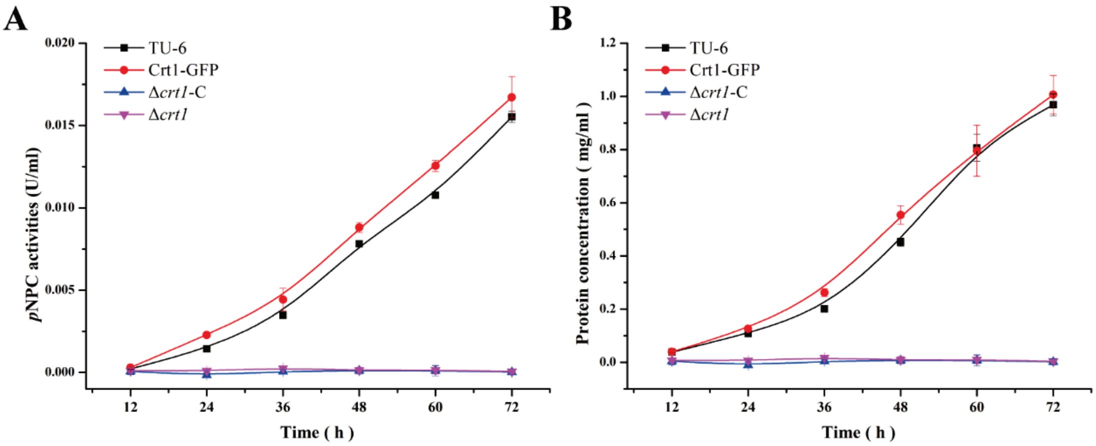

Figure S2. The *in situ* fused Crt1-GFP was functional for cellulase induction in TU-6 and *in situ* deletion of Crt1 C-terminus (Δ*crt1*-C) abolished the expression of cellulases in TU-6. Extracellular *p*NPC hydrolytic activities (A) and total protein concentration (B) of TU-6, Crt1-GFP, Δ*crt1*, and Δ*crt1*-C strain were determined and presented. Both of the strains were cultured in MA medium containing 1% (w/v) Avicel as the sole carbon source. Values are the mean of three biological replicates and error bars represent the Standard Deviation (SD) of these replicates.

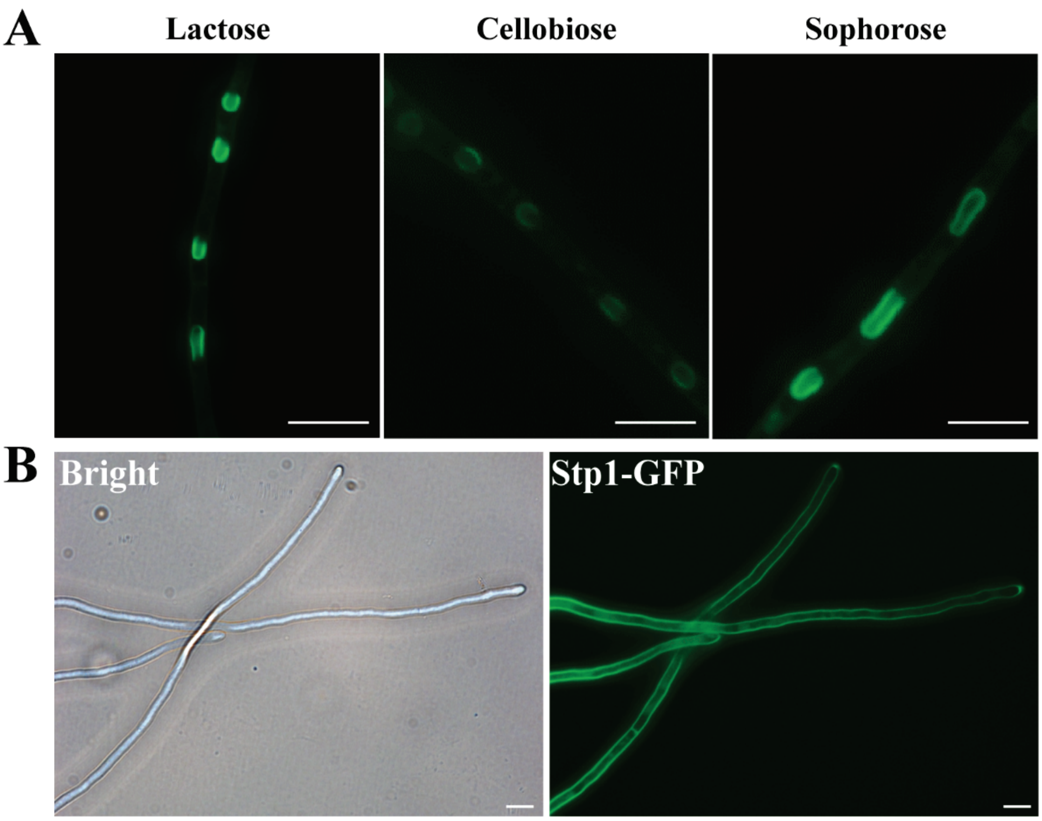

Figure S3. Localization analysis of Crt1-GFP and Stp1-GFP by fluorescence microscopy. (A) Subcellular localization analysis of Crt1-GFP on different carbon sources by fluorescence microscopy. Crt1-GFP strain was cultivated in MA medium containing 1% (w/v) lactose, 1% (w/v) cellobiose, and 1 mM sophorose as the sole carbon source for 12 h, respectively, and mycelia were collected for fluorescence microscopy analysis. Scale bar: 10 µm. (B) Subcellular localization analysis of Stp1-GFP by fluorescence microscopy. GFP was also fused to the C-terminal of Stp1 *in situ*. Stp1-GFP strain was cultivated in MA medium containing 1% (w/v) Avicel as the sole carbon source for 12 h and mycelia were collected for fluorescence microscopy analysis. The images shown are taken from one of at least two independent experiments. Scale bar: 10 µm.

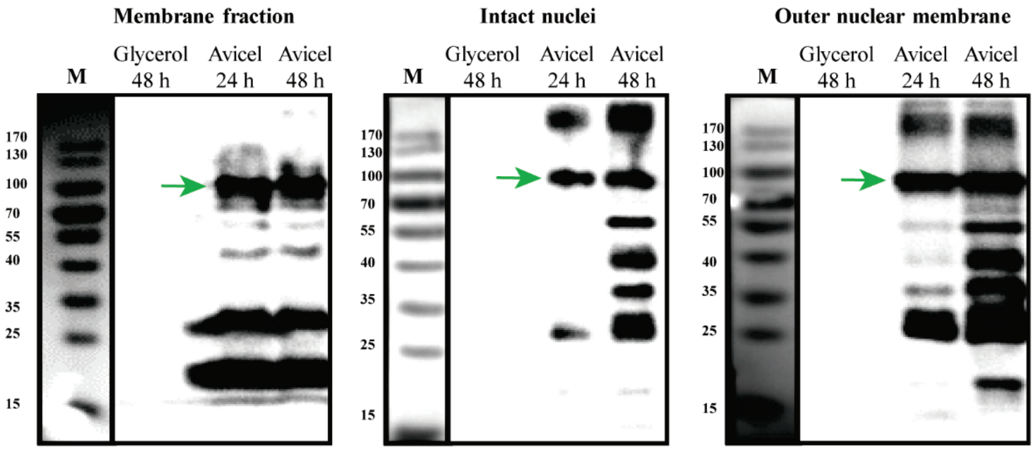

Figure S4. Western blot analysis of Crt1-GFP in different isolated cell fractions. Mycelia of Crt1-GFP strain was cultured in MA medium with 1% (w/v) Avicel and collected for the membrane fraction (left), intact nuclei fraction (middle), and outer nuclear membrane fraction (right) isolation as described in Material and Method section. A GFP monoclonal antibody was used for the detection of different cell fractions. The blot signal of Crt1-GFP was indicated by the green arrow.

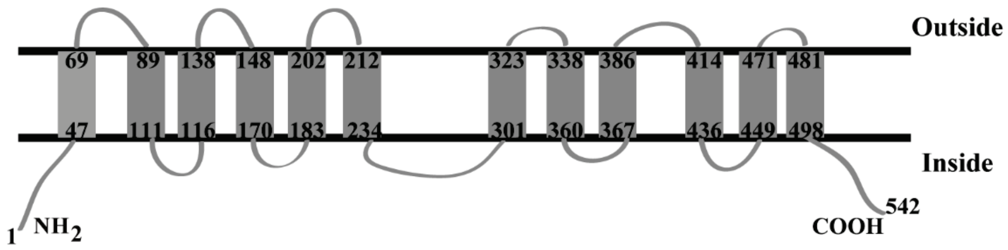

Figure S5. Secondary structure prediction of Crt1 by the TMHMM server. The transmembrane secondary structure of Crt1 was predicted by the TMHMM server (http://www.cbs.dtu.dk/services/TMHMM-2.0). The sections outside and inside plasma membrane and transmembrane sections were labeled in the figure.

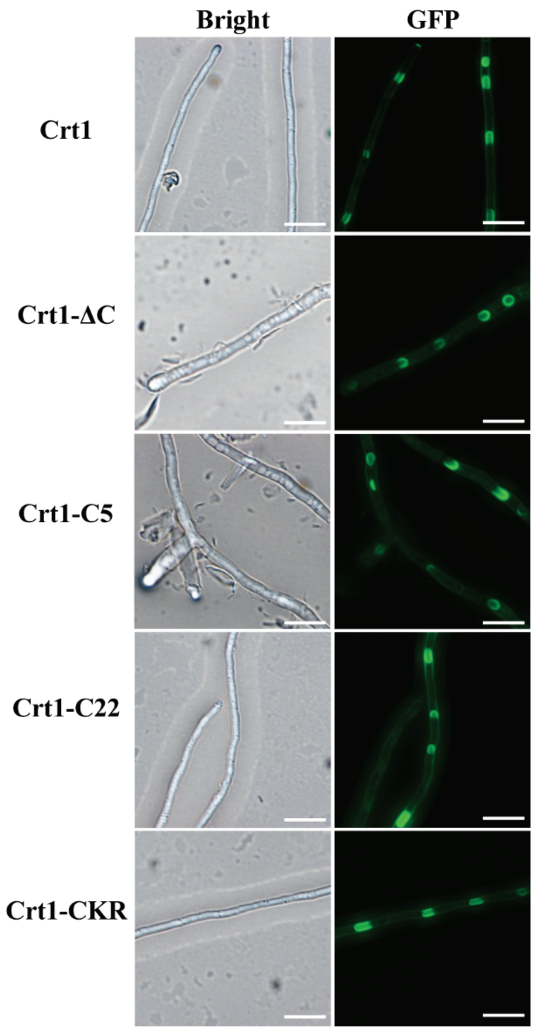

Figure S6. Fluorescence analysis of Crt1 and its mutants in various complementary strains. Intracellular localization of wild-type and various complemented Crt1 mutants were analyzed by fluorescence microscopy. All strains were cultivated in MA medium containing 1% (w/v) Avicel for 12 h. Scale bar: 10 µm.

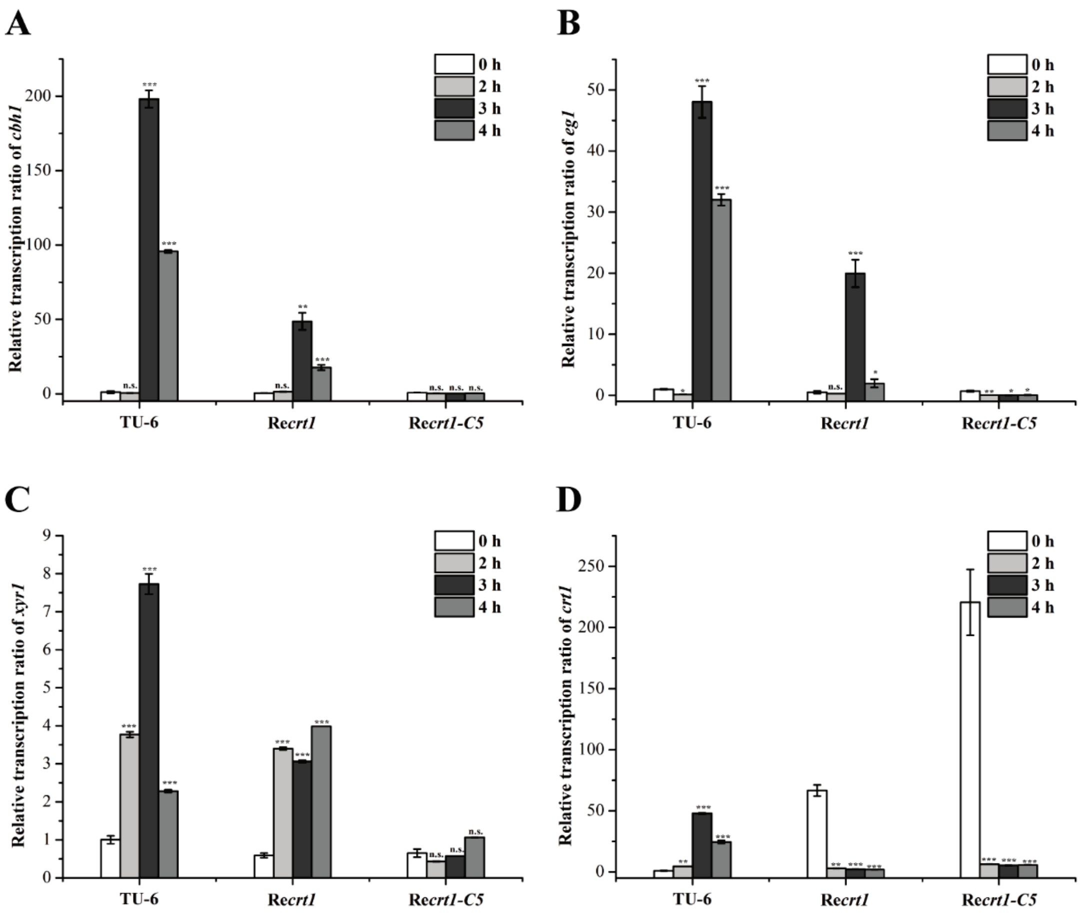

Figure S7. Overexpression of *crt1* did not advance the transcription of cellulase genes induced by Avicel. Relative transcription levels of cellulase genes (A, *cbh1*; B, *eg1*), *xyr1* (C), and *crt1* (D) were determined in the resting-cell system using 1% (w/v) Avicel as the sole carbon source. Values are the mean of three biological replicates and error bars represent the Standard Deviation (SD) of these replicates. The expression level of the actin gene was used as an endogenous control for all samples. Statistical significances of the target gene transcription relative to 0 h of each strain were determined using Student's t-test (n.s., $P > 0.05$; *, $P < 0.05$; **, $P<0.01$; ***, $P<0.001$).

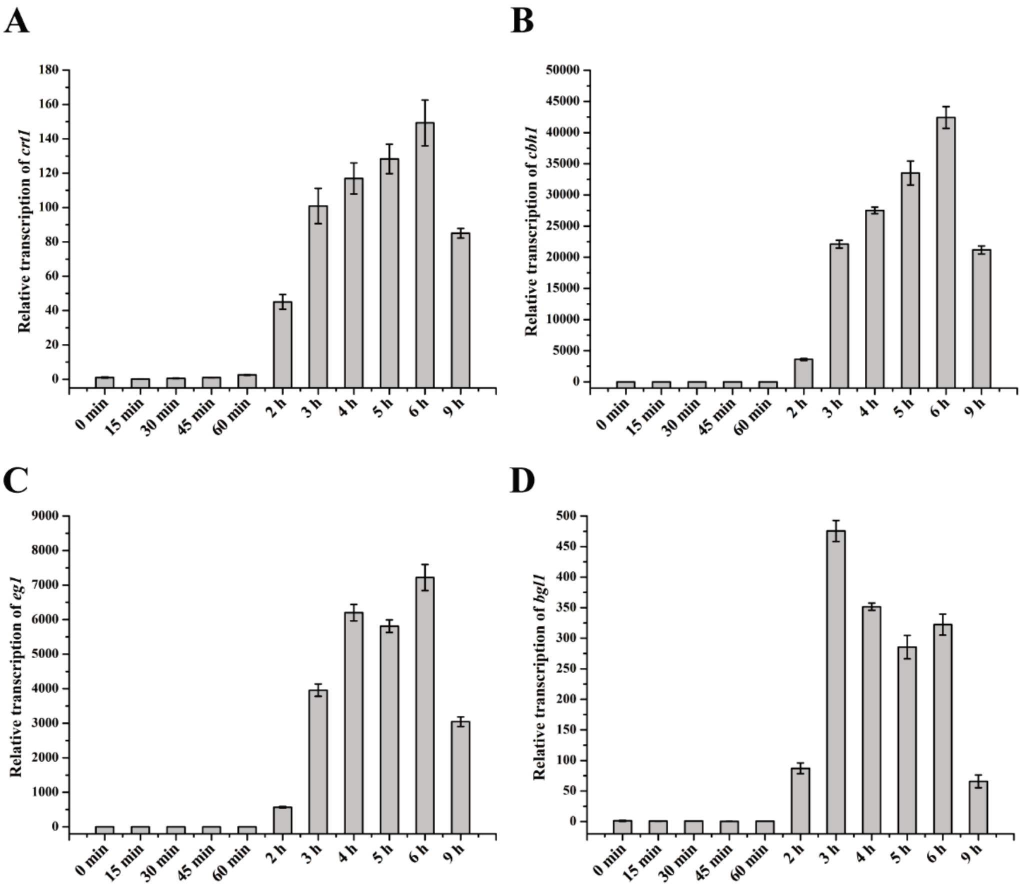

Figure S8. The transcription profile of *crt1* is similar to major cellulase genes upon induction by Avicel. The kinetic transcription levels of *crt1* (A), *cbh1* (B), *eg1* (C), *bgl1* (D) at the early stage of the induction process were analyzed by quantitative RT-PCR. All strains were cultivated in MA medium containing 1% (w/v) Avicel as the sole carbon source. Values are the mean of three biological replicates and error bars represent the Standard Deviation (SD) of these replicates.
